# Supplementary material for: Herbicidal Effects and Cellular Targets of Aqueous Extracts from Young Eucalyptus globulus Labill. Leaves
Source: Plants (Basel). 2021 Jun 7;10(6):1159. doi: 10.3390/plants10061159 (PMC8273694; doi:10.3390/plants10061159)
Supplement: Supplementary file 1 [file plants-10-01159-s001.zip › plants-1221045-supplementary.pdf]

Supplementary Materials

Herbicidal Effects and Cellular Targets of Aqueous Extracts from Young *Eucalyptus globulus* Labill. Leaves

Mafalda Pinto [1], Cristiano Soares [1], Maria Martins [1], Bruno Sousa [1], Inês Valente [2,3], Ruth Pereira [1] and Fernanda Fidalgo [1,*]

[1] GreenUPorto - Sustainable Agrifood Production Research Centre, Departamento de Biologia, Faculdade de Ciências, Universidade do Porto, Rua do Campo Alegre, 4169-007, Porto, Portugal
[2] REQUIMTE, LAQV, Departamento de Química e Bioquímica, Faculdade de Ciências, Universidade do Porto, Rua do Campo Alegre, 4169-007, Porto, Portugal
[3] REQUIMTE, LAQV, ICBAS, Instituto de Ciências Biomédicas Abel Salazar, Universidade do Porto, Rua Jorge Viterbo Ferreira, 228, 4050-313, Porto, Portugal
* Correspondence: ffidalgo@fc.up.pt

Table S1. Summary of repeated measures' ANOVA statistical data for the percentage of viable plants (% viable plants) treated with the extracts prepared with fresh leaves (FLE) and with dried leaves (DLE).

	Parameter	Time (weeks)	Time x Concentration
FLE	% viable plants	F (1.81, 34.4) = 29.2; $p \leq 0.001$	F (10.9, 34.4) = 14.9; $p \leq 0.001$
DLE	% viable plants	F (2.48, 49.7) = 44.7; $p \leq 0.001$	F (14.9, 49.7) = 20.8; $p \leq 0.001$

Table S2. Summary of one-way ANOVA statistical data for the percentage of viable plants (% viable plants) for each tested concentration of the extracts prepared with fresh leaves (FLE) and with dried leaves (DLE), as well as glyphosate (GLY), over the exposure period (5 weeks).

Situation	Concentration [% (v/v)]	% viable plants
FLE	0%	-
	12.5%	F (5, 17) = 0.700; $p > 0.05$
	25%	-
	50%	F (5, 17) = 0.542; $p > 0.05$
	75%	F (5, 17) = 2.47; $p > 0.05$
	100%	F (5, 17) = 1.76; $p > 0.05$
DLE	0%	-
	12.5%	-
	25%	F (5, 18) = 0.600; $p > 0.05$
	50%	F (5, 18) = 0.600; $p > 0.05$
	75%	-
	100%	F (5, 18) = 26.9; $p \leq 0.001$
GLY	-	F (5, 17) = 14.2; $p \leq 0.001$

Table S3. Summary of ANOVA statistical data for the percentage of viable plants (% viable plants) of the purslane plants treated with increasing concentrations of the extracts prepared with fresh leaves (FLE) and with dried leaves (DLE).

Parameter	FLE	DLE
% viable plants	F (6, 20) = 78.1; $p \leq 0.001$	F (6, 21) = 101; $p \leq 0.001$

Table S4. Summary of the two-way ANOVA statistical data for the shoot and root length and fresh biomass of the purslane plants treated with increasing concentrations of the extracts prepared with fresh leaves (FLE) and with dried leaves (DLE).

Parameter	Type of extract	Concentration	Type of extract × Concentration
Shoot length	F (1, 36) = 20.8; $p \leq 0.001$	F (6, 36) = 115; $p \leq 0.001$	F (6, 36) = 2.39; $p \leq 0.05$
Shoot biomass	F (1, 33) = 90.7; $p \leq 0.001$	F (6, 33) = 116; $p \leq 0.001$	F (6, 33) = 14.6; $p \leq 0.001$
Root length	F (1, 38) = 8.17; $p \leq 0.05$	F (6, 38) = 133; $p \leq 0.001$	F (6, 38) = 23.1; $p \leq 0.001$
Root biomass	F (1, 33) = 2.02; $p > 0.05$	F (6, 33) = 42.3; $p \leq 0.001$	F (6, 33) = 11.5; $p \leq 0.001$

Table S5. Summary of one-way ANOVA statistical data obtained for the shoots and roots of purslane plants treated with the extract prepared with dried leaves (DLE) at 75% (v/v) and 100% (v/v).

Parameter	Shoots	Roots
H_2O_2	F (2, 9) = 24.5; $p \leq 0.001$	F (2, 7) = 116; $p \leq 0.001$
MDA	F (2, 8) = 2.58; $p > 0.05$	F (2, 6) = 15.8; $p \leq 0.05$
Total sugars	F (2, 7) = 22.8; $p \leq 0.001$	F (2, 7) = 16.9; $p \leq 0.05$
Free amino acids	F (2, 6) = 34.6; $p \leq 0.001$	F (2, 6) = 11.4; $p \leq 0.05$
Proline	F (2, 7) = 4.42; $p > 0.05$	-
Proteins	F (2, 9) = 28.2; $p \leq 0.001$	F (2, 13) = 12.6; $p \leq 0.001$
Total chlorophylls	F (2, 6) = 8.10; $p \leq 0.05$	-

Carotenoids	F (2, 6) = 13.1; $p \leq 0.05$	-
GS	F (2, 8) = 1.53; $p > 0.05$	F (2, 6) = 27.9; $p \leq 0.001$
NR	F (2, 6) = 65.5; $p \leq 0.001$	F (2, 6) = 1.33; $p > 0.05$
